# Covered self-expandable metal stent for hemostasis of ruptured pseudoaneurysm caused by partial stent-in-stent method

**DOI:** 10.1055/a-2219-2756

**Published:** 2023-12-21

**Authors:** Hideaki Kazumori, Masaki Onoe, Satoshi Ooya, Yasuhiko Ohno, Kousaku Kawashima

**Affiliations:** 1 13838 Department of Gastroenterology, Matsue Seikyo General Hospital, Matsue, Japan; 2 Department of Internal Medicine II, Shimane University, Izumo, Japan

Placement of a covered self-expandable metal stent (CSEMS) is useful for hemostasis in patients with hemobilia 1 2 . Following SEMS placement with a partial stent-in-stent (PSIS) method, an SEMS is not generally used for hilar bile duct hemostatsis as this type of stent may cause contralateral biliary obstruction. This is the first reported case of pseudoaneurysm rupture caused by PSIS, in which hemostasis was subsequently achieved using a CSEMS.

An 80-year-old man with a hilar cholangiocarcinoma underwent endoscopic retrograde cholangiopancreatography (ERCP), which revealed hilar biliary obstruction ( Fig. 1 **a** ). Bilateral biliary drainage using a PSIS method was planned. Following SEMS deployment into the left hepatic duct, another stent was inserted into the right hepatic duct in a stent-in-stent manner ( Fig. 1 **b** ). One month later, cholangitis developed and a plastic stent was placed through the SEMS in the right hepatic duct ( Fig. 1 **c** ).

**Fig. 1:**
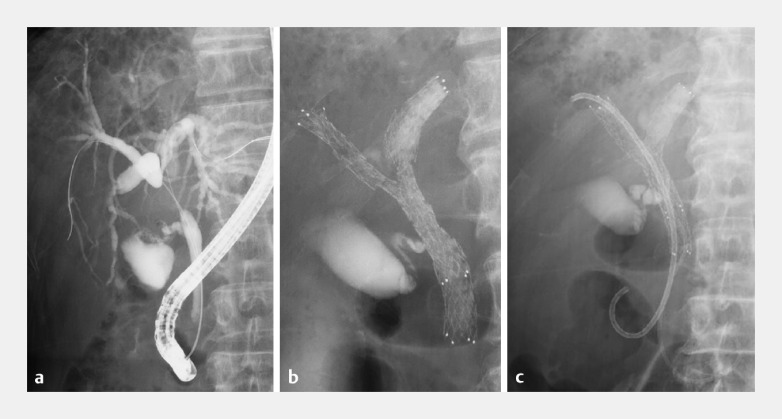
Endoscopic retrograde cholangiopancreatography images. **a** Hilar biliary obstruction. **b** Self-expandable metal stents (SEMSs) (Zeostent V; Zeon Medical, Inc., Tokyo, Japan) were placed using a partial stent-in-stent method (left: diameter 10 mm, length 6 cm; right: diameter 10 mm, length 8 cm). **c** A plastic stent (7 Fr, length 8 cm, without flap) was placed through the SEMS into the right hepatic duct.

Cholangitis developed again after 1 month and ERCP showed blood around the plastic stent; however, when the plastic stent was removed, pulsating bleeding caused loss of view ( Fig. 2 **a** , Video 1 ). Hemostasis by CSEMS placement, followed by its removal to prevent obstruction of the left bile duct, was planned.

**Fig. 2:**
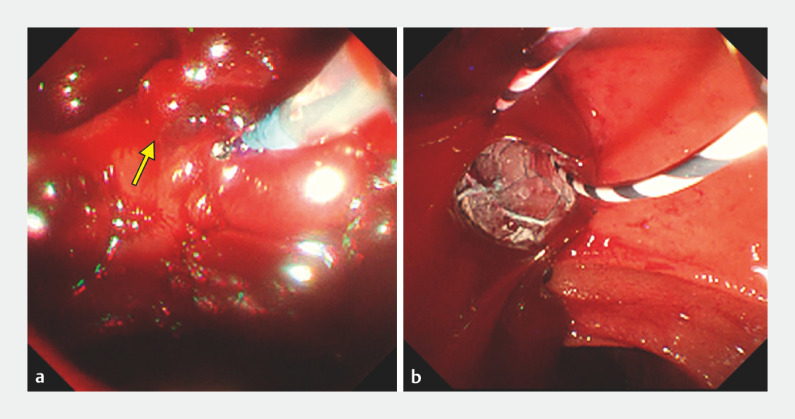
Endoscopy images. **a** Pulsating bleeding from the duodenal papilla (arrow) caused by pseudoaneurysm rupture. **b** After placement of a covered self-expandable metal stent (Niti-S SUPREMO, diameter 10 mm, length 8 cm; Century Medical, Inc., Tokyo, Japan), hemostasis was achieved.

When the plastic stent was removed, pulsating bleeding occurred. After placement of a covered self-expandable metal stent (CSEMS), hemostasis was successfully achieved. The CSEMS was removed 2 weeks later.Video 1

A CSEMS was inserted through the SEMS in the right hepatic duct and extended from the duct to the papilla. The field of view was secured ( Fig. 2 **b** ). Angiography was performed and revealed a pseudoaneurysm of the right hepatic artery involving the SEMS, with no further extravasation from the pseudoaneurysm noted ( Fig. 3 **a** ). The angiography findings confirmed that the bleeding was from the pseudoaneurysm and that placement of the CSEMS had successfully achieved hemostasis. The pseudoaneurysm was then treated with coil embolization ( Fig. 3 **b** ) and the CSEMS was removed after 2 weeks.

**Fig. 3:**
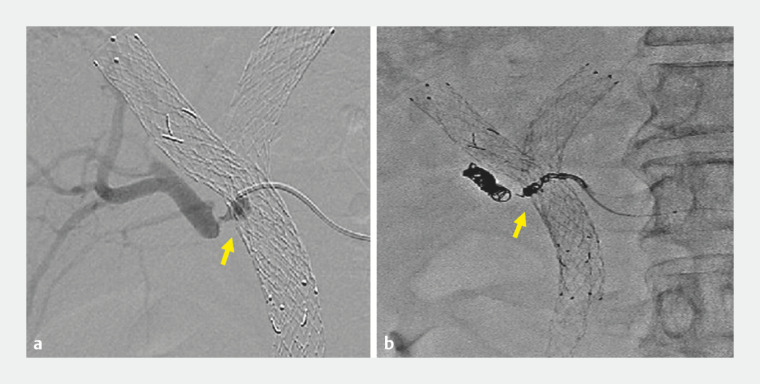
Angiography images. **a** Pseudoaneurysm (arrow) in the right hepatic artery. **b** Coil embolization treatment (arrow) was performed.

Pseudoaneurysm rupture is a life-threatening condition, with emergency hemostasis necessary as a life-saving procedure. In the present case, primary hemostasis was achieved as planned by use of a CSEMS following a PSIS method.

Endoscopy_UCTN_Code_TTT_1AR_2AZ

## Notes

Endoscopy E-Videos https://eref.thieme.de/e-videos
*E-Videos* is an open access online section of the journal *Endoscopy* , reporting on interesting cases and new techniques in gastroenterological endoscopy. All papers include a high-quality video and are published with a Creative Commons CC-BY license. Endoscopy E-Videos qualify for HINARI discounts and waivers and eligibility is automatically checked during the submission process. We grant 100% waivers to articles whose corresponding authors are based in Group A countries and 50% waivers to those who are based in Group B countries as classified by Research4Life (see: https://www.research4life.org/access/eligibility/ ). This section has its own submission website at https://mc.manuscriptcentral.com/e-videos .
